# Endometrial Neuroendocrine Carcinoma With Widespread Metastases: Diagnostic and Therapeutic Challenges in a Rare Malignancy

**DOI:** 10.7759/cureus.105406

**Published:** 2026-03-17

**Authors:** Anjiya Aswani, Oluwaseyi A Akinyooye, Meena Barakam, Anuradha Sakhuja, Marianne Greene

**Affiliations:** 1 Internal Medicine, Ross University School of Medicine, Bridgetown, BRB; 2 Obstetrics and Gynecology, Ross University School of Medicine, Bridgetown, BRB; 3 Internal Medicine, Mount Sinai Hospital, Chicago, USA; 4 Pathology, Mount Sinai Hospital, Chicago, USA

**Keywords:** endometrial cancers, histopathology, multimodal therapy, neuroendocrine tumors, oncology pathology, rare gynecologic malignancy

## Abstract

Endometrial neuroendocrine carcinomas (NECs) are rare, aggressive malignancies with early metastatic potential and poor prognosis. We present the case of a 54-year-old woman who presented with thoracic back pain and lower extremity paresthesia. Imaging revealed a T1 compression fracture, additional spinal metastases, liver and lung lesions, a thickened endometrium, and a left adnexal mass. Tumor markers, including CA-125, CA 19-9, and CEA, were markedly elevated. Thoracic spine MRI confirmed spinal stenosis and metastatic involvement. She underwent urgent T1 laminectomy with C5-T3 fusion, followed by systemic therapy with carboplatin/paclitaxel plus pembrolizumab, and later transitioned to carboplatin/etoposide. Treatment was complicated by *Clostridioides difficile* infection and chemotherapy-induced leukopenia, and she received palliative spinal radiation for symptom control. Histopathology confirmed small-cell NEC with squamous differentiation; expert review favored a poorly differentiated/dedifferentiated endometrial carcinoma with neuroendocrine and squamoid features. This case illustrates an atypical initial presentation with neurologic deficits from spinal metastases, emphasizing the need for prompt recognition and multidisciplinary management.

## Introduction

Neuroendocrine tumors (NETs) of the endometrium are exceedingly rare, representing less than 1% of all endometrial malignancies, and are frequently associated with aggressive clinical behavior and poor outcomes [1-3]. They are broadly classified into small-cell neuroendocrine carcinoma (SCNEC) and large-cell neuroendocrine carcinoma (LCNEC), although neuroendocrine differentiation can also occur in mixed endometrial carcinomas [4,5]. Patients often present at an advanced stage with extrauterine disease, reflecting the highly aggressive biology of these tumors [6].

Diagnosis is challenging due to morphologic overlap with other high-grade endometrial carcinomas, including high-grade endometrioid carcinoma, serous carcinoma, and undifferentiated carcinoma. Immunohistochemical markers such as synaptophysin, chromogranin, and CD56 are essential for confirming neuroendocrine differentiation, while the Ki-67 proliferative index assists in grading [7]. In addition, evolving molecular classification frameworks for endometrial carcinoma have provided further insights into tumor biology and prognostic stratification, highlighting the importance of integrating histopathologic, immunohistochemical, and molecular findings in the evaluation of these rare tumors.

There are no established consensus guidelines for management because of the rarity of these tumors. Current treatment strategies are largely extrapolated from small-cell lung carcinoma protocols, including platinum-based chemotherapy (carboplatin/etoposide) and, in selected cases, multimodal approaches incorporating surgery and radiation [8,9]. Despite aggressive treatment, prognosis remains poor, with most patients experiencing early recurrence or rapid progression [3,6].

We present the case of a 54-year-old woman with metastatic endometrial neuroendocrine carcinoma (NEC) who initially presented with back pain and neurological symptoms and was later found to have widespread disease involving the spine, lungs, liver, and adnexa. This case highlights the diagnostic complexity, therapeutic challenges, and aggressive clinical course of endometrial NETs, underscoring the need for improved recognition and reporting to optimize future management strategies.

## Case presentation

A 54-year-old woman (G0P0) with a history of depression, managed with fluoxetine, presented with two weeks of thoracic back pain radiating to her legs, accompanied by paresthesia. She denied trauma, weight loss, or substance use. On examination, she had midline thoracic and paraspinal tenderness, with intact motor strength at presentation. Laboratory studies revealed elevated alkaline phosphatase, elevated D-dimer, and microcytic anemia (Table 1).

**Table 1:** Abnormal laboratory findings on initial evaluation All values are from the initial laboratory assessment. Elevated D-dimer and alkaline phosphatase levels, along with microcytic indices (low mean corpuscular volume and mean corpuscular hemoglobin), were noted.

Laboratory study	Result	Reference range
Alkaline phosphatase	112 U/L	34-104 U/L
D-dimer	7,360 ng/mL	0-500 ng/mL
Mean corpuscular volume	73.7 fL	80-100 fL
Mean corpuscular hemoglobin	24.4 pg	26-34 pg

**Figure 1:**
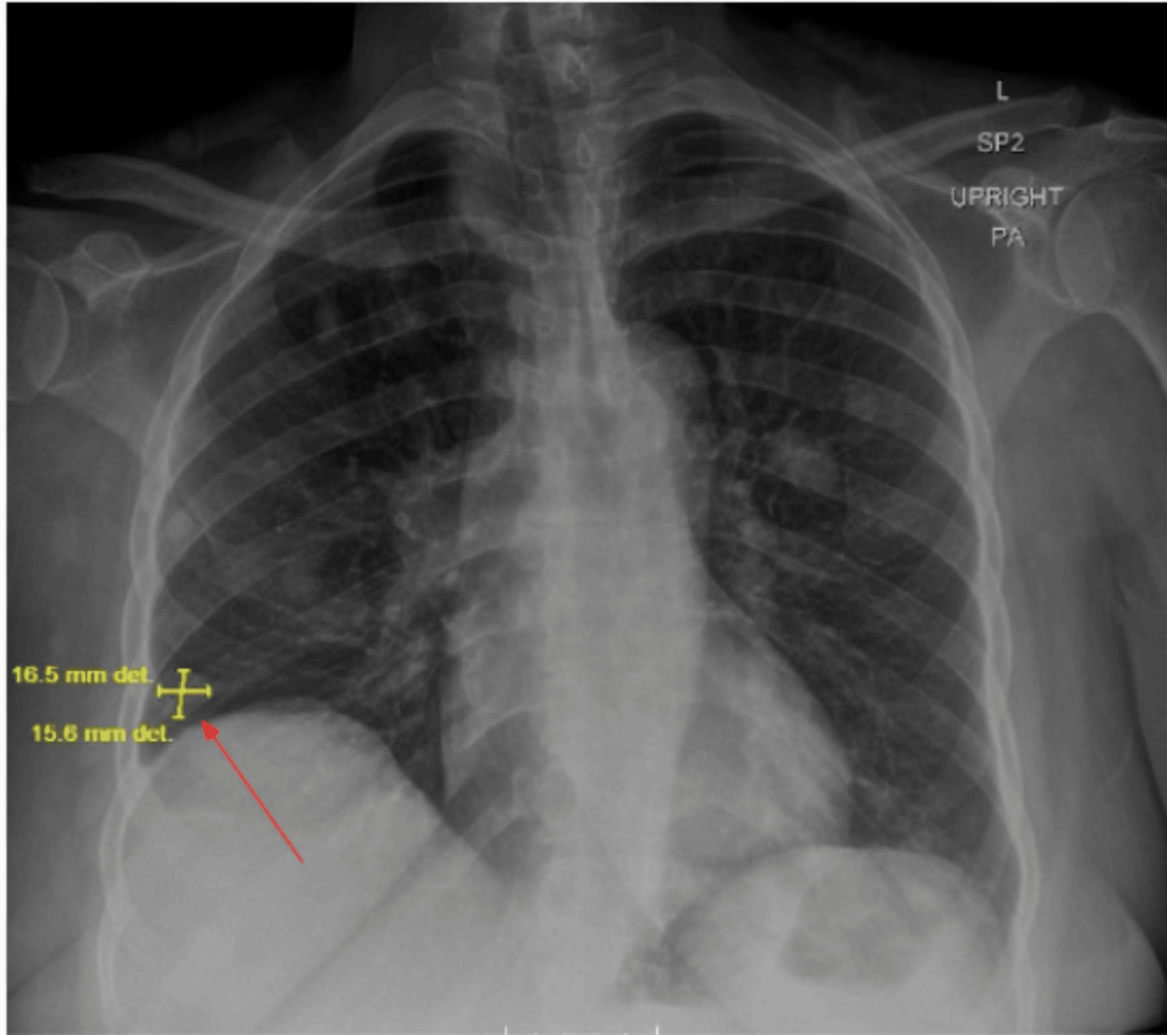
Chest radiograph showing multiple mass-like densities in the right lung A representative nodular density in the right lower lobe measures approximately 1.7 × 1.6 cm (thin red arrow).

Initial imaging with CT of the chest, abdomen, and pelvis revealed a T1 compression fracture, lytic lesions at T6 and T8, and metastatic involvement of the lungs and liver. Pelvic findings included a markedly thickened endometrium and a left adnexal mass, raising concern for a primary gynecologic malignancy. Chest radiography demonstrated multiple nodular densities in the right lung (Figure 1).

**Figure 2:**
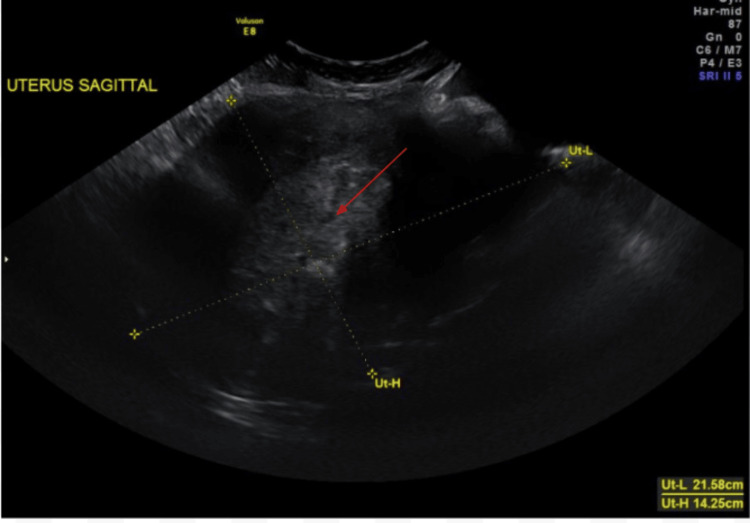
Pelvic ultrasound showing an enlarged fibroid uterus with numerous fibroids and a markedly thickened, heterogeneous endometrium measuring 4.8 cm (thin red arrow)

Pelvic ultrasound confirmed an enlarged fibroid uterus with a markedly thickened, heterogeneous endometrium measuring 4.8 cm, highly suspicious for a primary endometrial malignancy, along with a large left ovarian cystic mass concerning for adnexal involvement (Figure 2). Tumor markers were notable for elevated CA-125, CA 19-9, and CEA (Table 2). 

**Table 2:** Serum tumor marker levels at diagnosis Markedly elevated CA-125, CA 19-9, and CEA levels were noted, suggesting a possible malignant or inflammatory process.

Marker	Result	Reference range
Inhibin A	6 IU/L	1-10 IU/L
Inhibin B	<10 pg/mL	1-80 pg/mL
CA 15-3	32.4 U/mL	0-30 U/mL
Alpha-fetoprotein	1.9 ng/mL	0-9.0 ng/mL
CA-125	315 U/mL	<35 U/mL
CA 19-9	501 U/mL	<37 U/mL
CEA	11.8 ng/mL	<3 ng/mL (nonsmoker)

**Figure 3:**
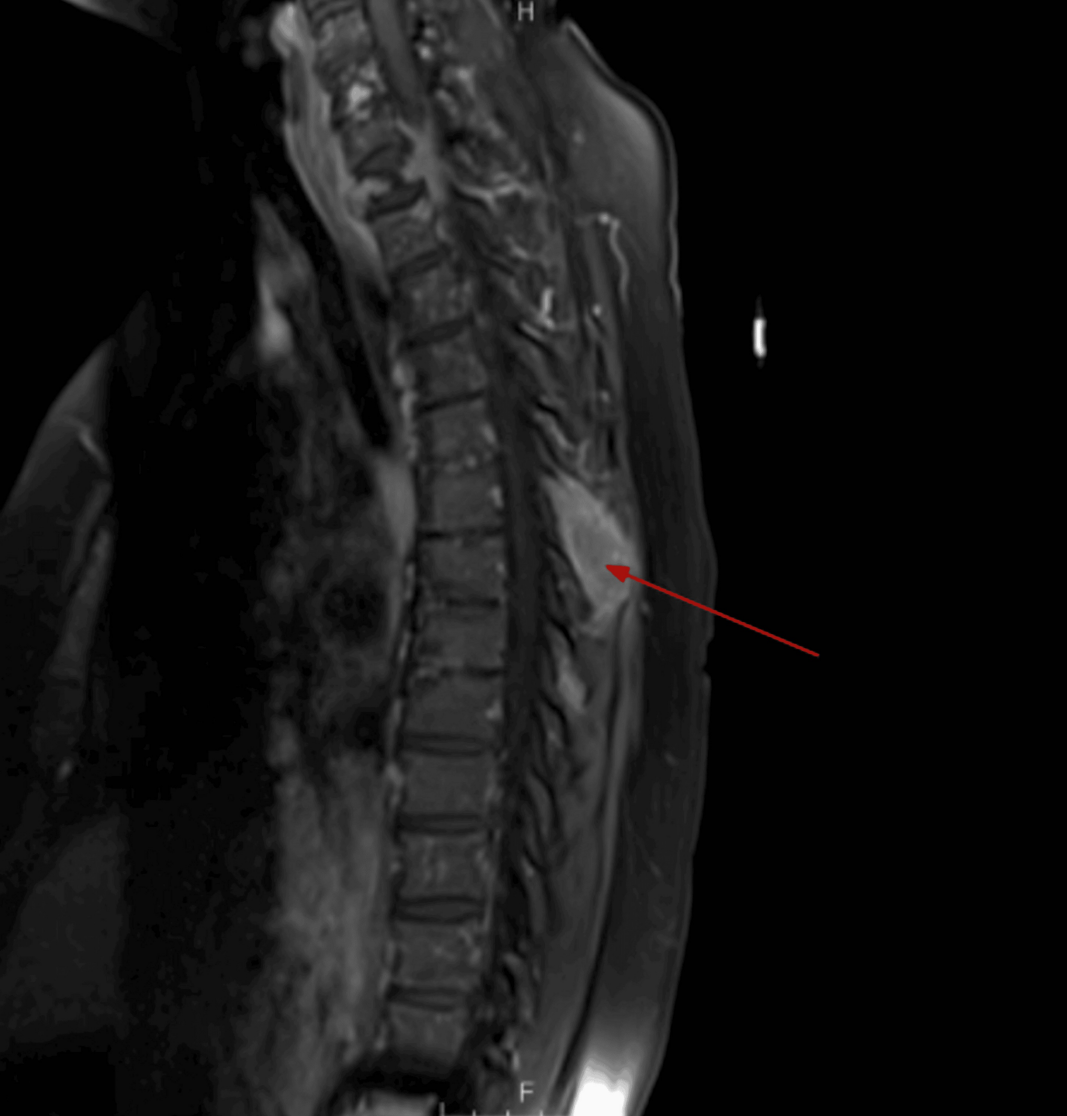
Sagittal T1 MRI showing mild vertebral retropulsion, possible posterior epidural involvement, and moderate spinal canal stenosis

Thoracic spine MRI demonstrated approximately 50% compression of T1 with post-contrast enhancement, mild retropulsion, possible posterior epidural involvement, and additional metastatic lesions at T3, T6, and T8 (Figure 3). Within three days of admission, the patient experienced progressive lower extremity weakness. Neurosurgery performed urgent decompression, including T1 laminectomy and C5-T3 fusion, with postoperative stabilization.

T1 MRI demonstrated mild vertebral retropulsion and possible posterior epidural space involvement, with at least moderate spinal canal stenosis at this level. An expansile mass with post-contrast enhancement involved the spinous process of T6, and an additional, more subtle enhancing metastatic lesion was present in the spinous process of T8.

**Figure 4:**
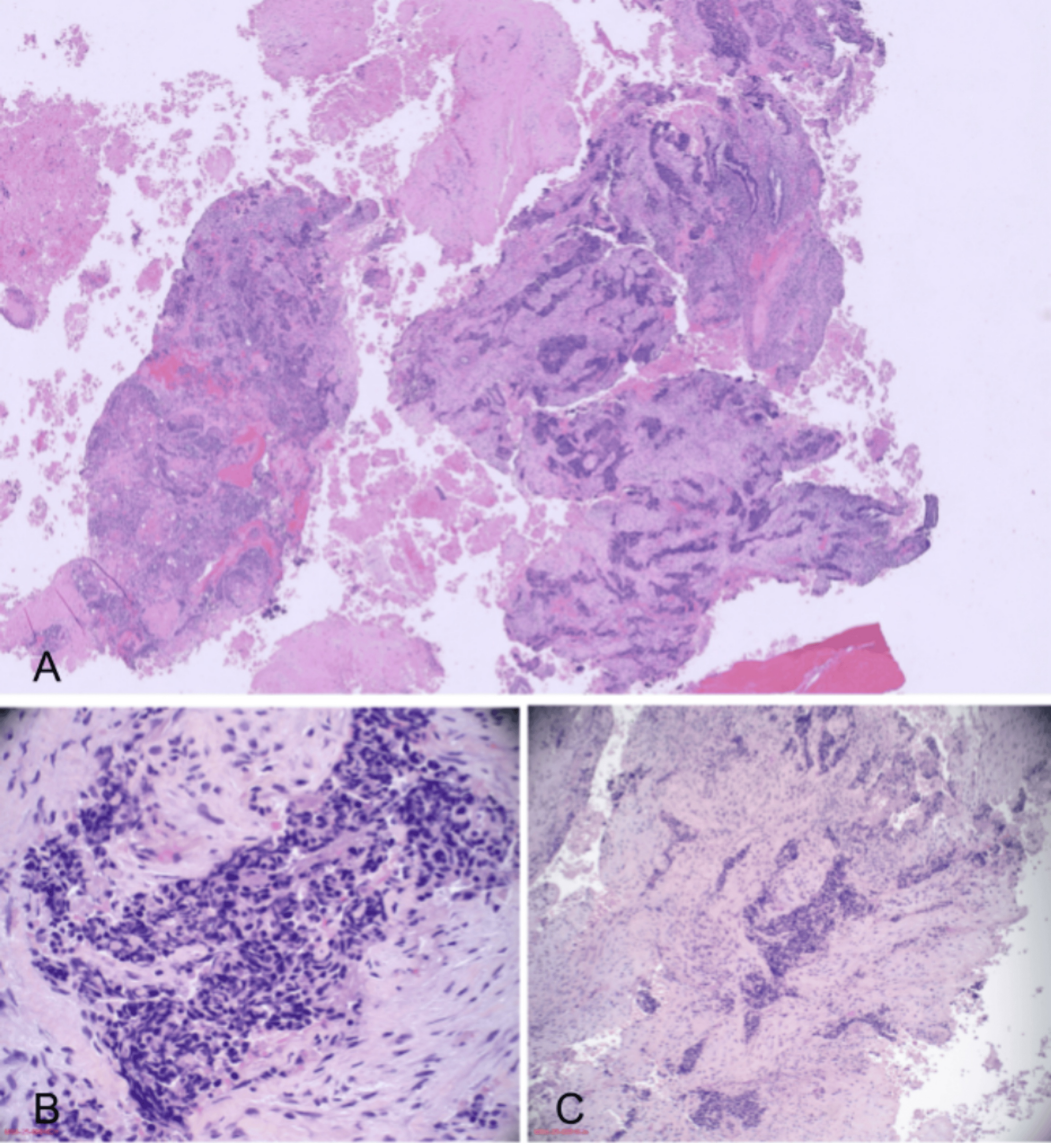
H&E staining of the endometrial tumor (A) Very low-power view (2×) showing tumor architecture within the endometrial tissue. (B) Low-power view (20×) demonstrating sheets of tumor cells with neuroendocrine differentiation. (C) High-power view (40×) highlighting characteristic small hyperchromatic cells with scant cytoplasm and nuclear molding.

**Figure 5:**
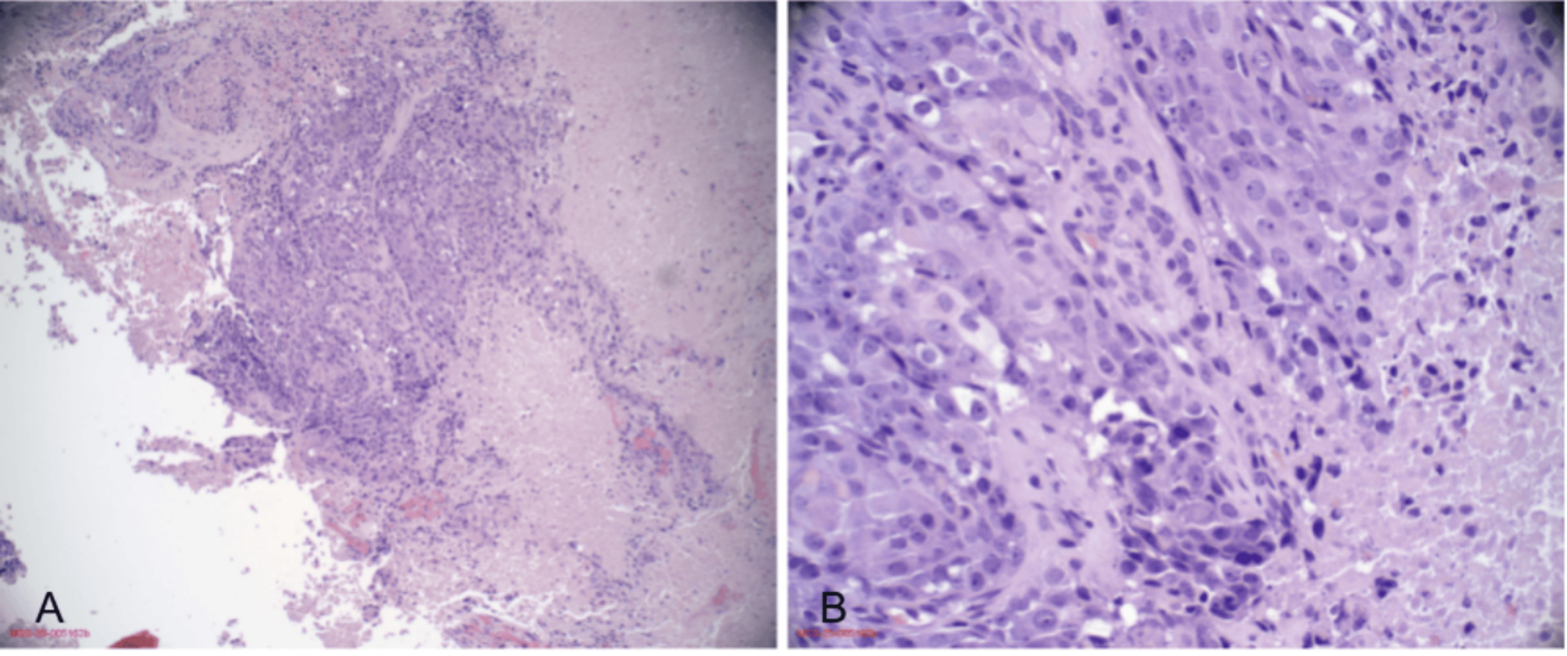
H&E staining of the endometrial tumor showing focal squamous differentiation (A) 20× view showing areas of squamous differentiation within the tumor. (B) 40× view highlighting keratinization and squamoid cellular morphology.

**Figure 6:**
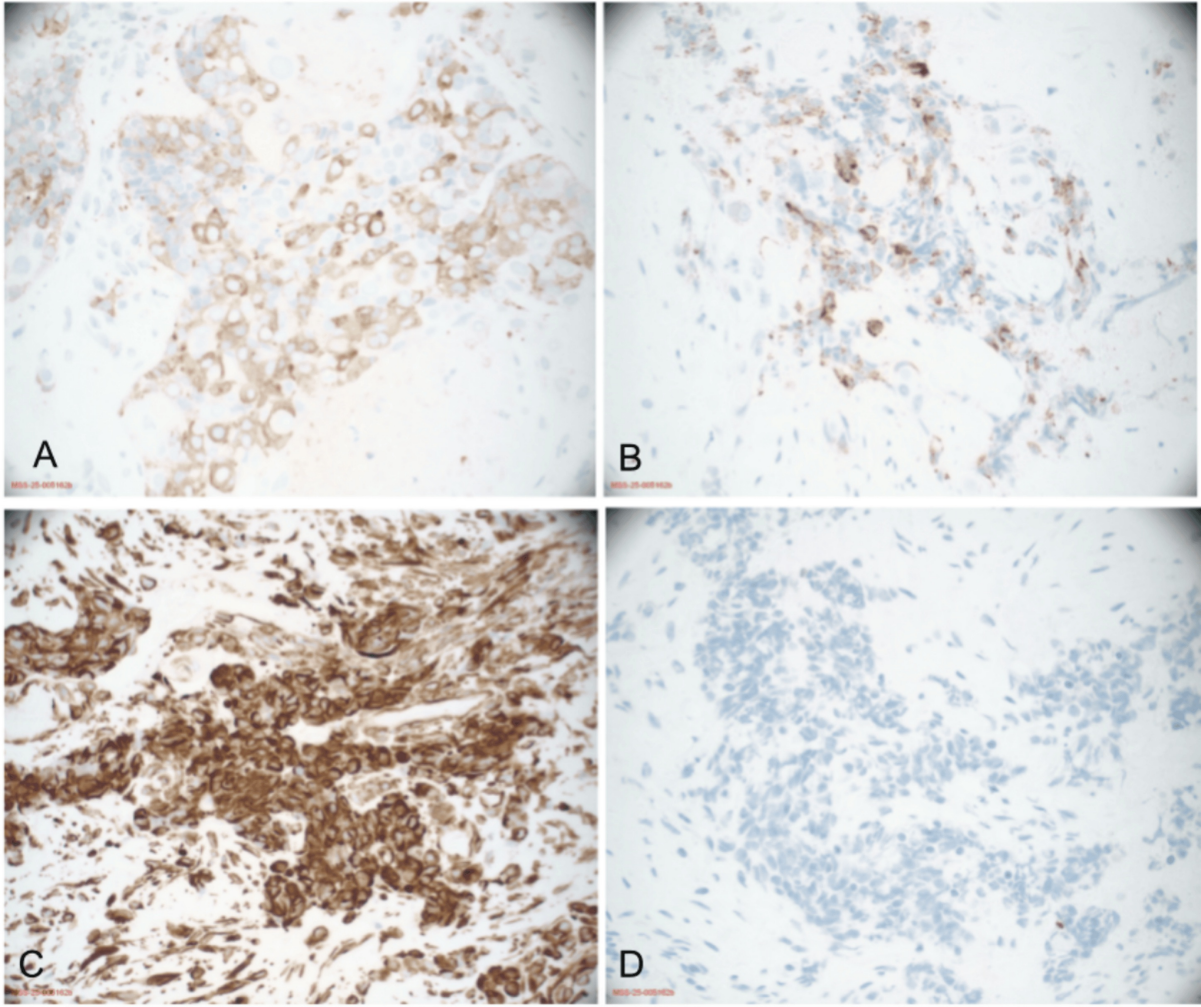
Immunohistochemical staining demonstrating neuroendocrine differentiation (A) Synaptophysin positivity (40×). (B) Chromogranin positivity (40×). (C) Vimentin positivity (40×). (D) p63 highlighting squamoid differentiation.

**Figure 7:**
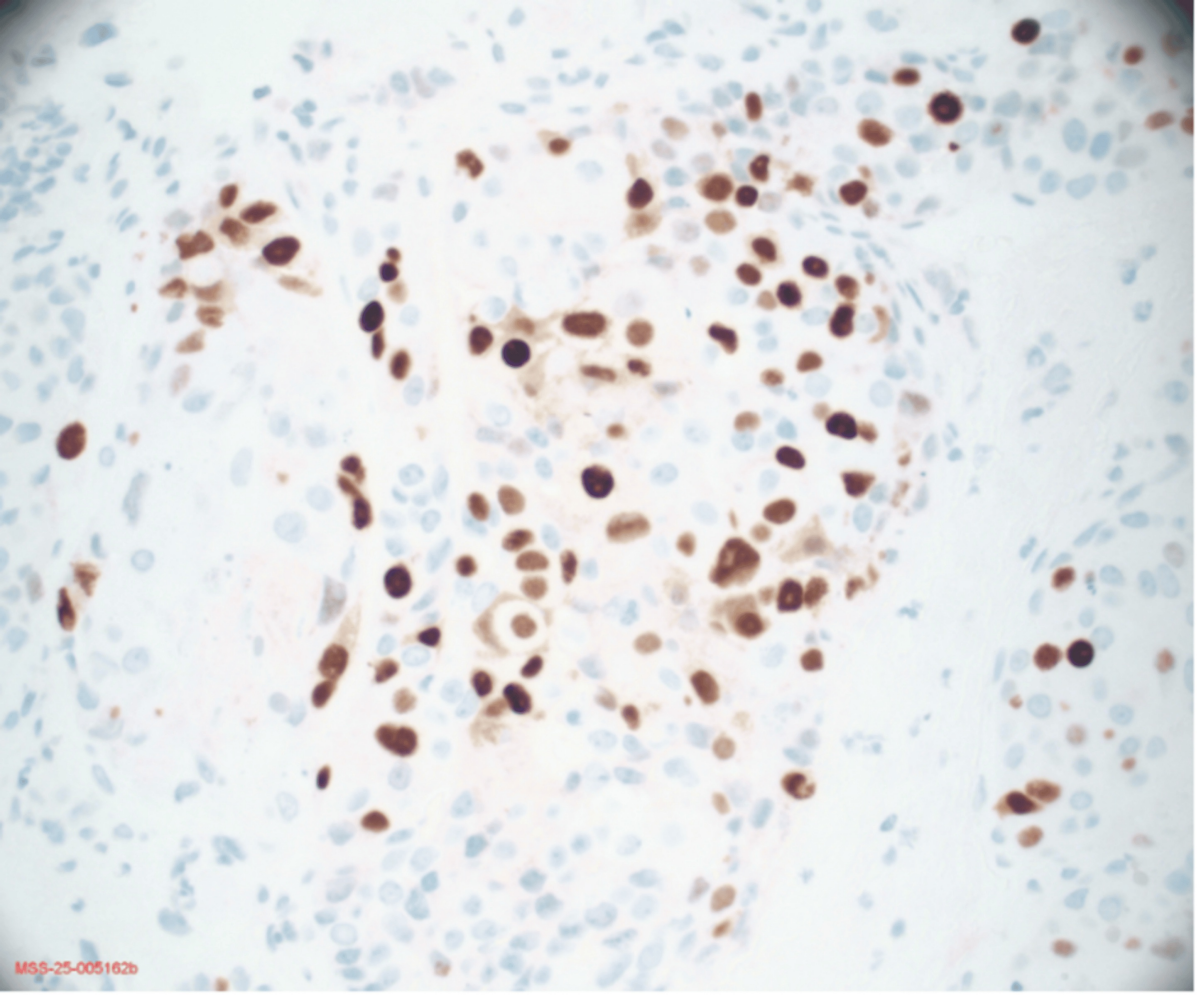
Immunohistochemical staining demonstrating squamous differentiation within the tumor Positive p63 staining is observed in the squamous component (40×).

Given the multifocal metastatic disease, tissue sampling was pursued. Endometrial curettings demonstrated SCNEC with focal squamous differentiation (Figure 4, Figure 5). Immunohistochemical staining revealed strong positivity for synaptophysin, chromogranin, and CD56, with a high proliferative index (Ki-67 > 80%). The neuroendocrine component was positive for synaptophysin, chromogranin, CD56, vimentin, and AE1/AE3 (Figure 6). Areas of squamous differentiation were positive for neuron-specific enolase and p63 (Figure 7), with patchy staining for p40, p63, and p16.

**Figure 8:**
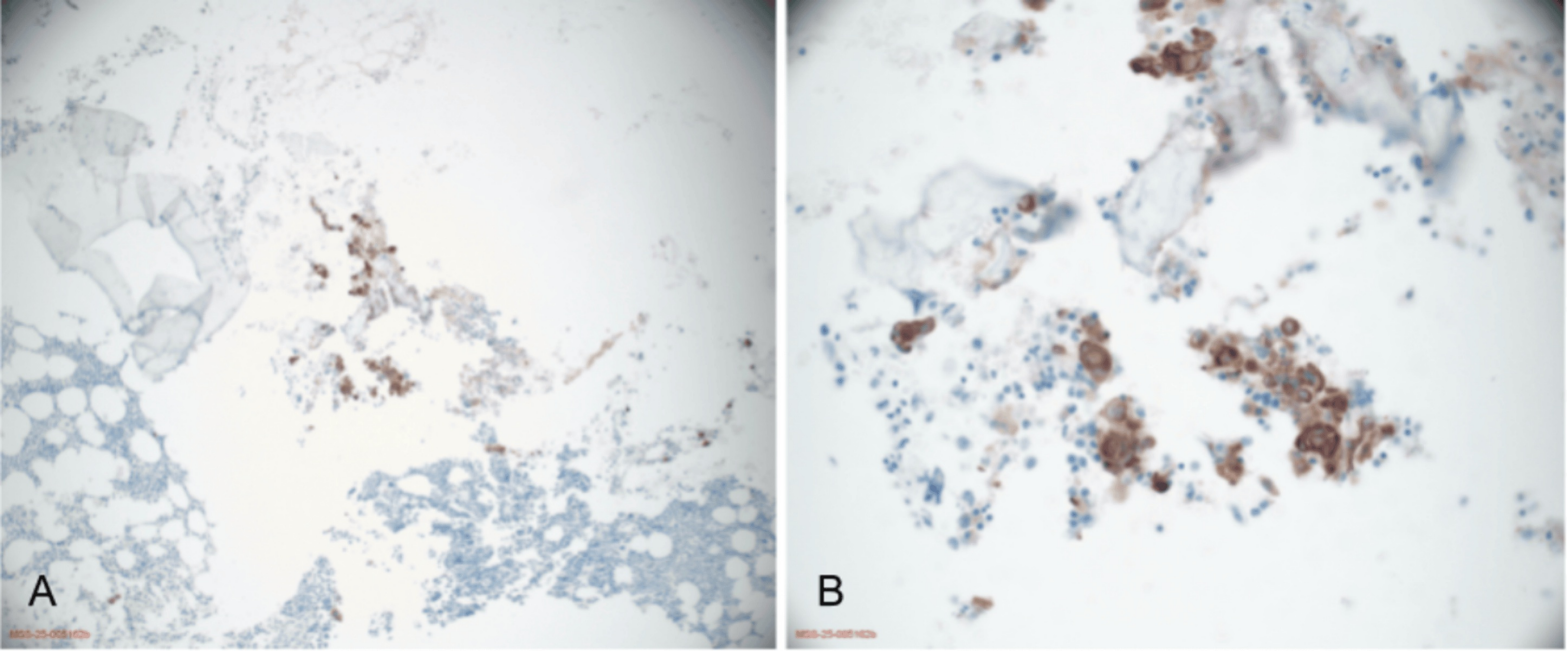
Biopsy of thoracic T1 vertebral lesion (bone and bone marrow) confirming metastatic NEC (A) AE1/AE3 immunostaining demonstrating cytokeratin positivity in metastatic carcinoma cells within bone marrow. (B) High-power view (40×) showing metastatic tumor infiltration of thoracic vertebral bone. NEC, neuroendocrine carcinoma

A biopsy of the T1 vertebral lesion confirmed metastatic NEC (Figure 8), while a right inguinal lymph node biopsy revealed metastatic poorly differentiated carcinoma consistent with endometrial origin. Expert consultation at Memorial Sloan Kettering Cancer Center favored a diagnosis of poorly differentiated or dedifferentiated endometrial carcinoma with neuroendocrine and squamoid features, accompanied by reduced MLH1 and PMS2 expression. 

Systemic therapy was initiated with carboplatin/paclitaxel plus pembrolizumab and later transitioned to carboplatin/etoposide with allopurinol prophylaxis for tumor lysis syndrome. During treatment, she developed *Clostridioides difficile* infection, managed with oral vancomycin, and chemotherapy-induced leukopenia, managed with granulocyte-colony stimulating factor. Palliative spinal radiation was administered for pain control. The patient was subsequently discharged to a rehabilitation facility with plans to continue chemotherapy and radiation as outpatient therapy.

## Discussion

Endometrial NECs are rare but highly aggressive malignancies characterized by early metastasis and poor prognosis [1,2]. Both SCNEC and LCNEC variants exhibit rapid progression and are often diagnosed at advanced stages with extrauterine spread, commonly to the lungs, liver, and bone, as in our patient [3,7]. Diagnosis is challenging due to morphologic overlap with other high-grade endometrial tumors. Immunohistochemistry remains essential, with synaptophysin, chromogranin, CD56, and Ki-67 confirming neuroendocrine differentiation and proliferation [5,6]. Accurate histologic classification is crucial for guiding therapy, which is largely based on protocols developed for small-cell lung cancer [8].

Distinguishing primary SCNEC from poorly differentiated or dedifferentiated endometrial carcinoma with neuroendocrine differentiation can be challenging because of overlapping morphologic and immunohistochemical features [4,5]. Dedifferentiated endometrial carcinoma typically contains a well-differentiated endometrioid component alongside an undifferentiated or poorly differentiated component. In contrast, true NECs demonstrate diffuse neuroendocrine morphology and strong expression of markers such as synaptophysin, chromogranin, and CD56 [5,6]. In our case, expert pathologic consultation favored a poorly differentiated or dedifferentiated endometrial carcinoma with neuroendocrine and squamoid features, highlighting the diagnostic complexity of these tumors.

Recent advances in molecular classification of endometrial carcinoma, including POLE-ultramutated, mismatch repair-deficient, p53-abnormal, and no specific molecular profile subtypes, have further refined diagnostic and prognostic assessment [2,3]. The reduced expression of MLH1 and PMS2 observed in this case suggests mismatch repair deficiency, which may have therapeutic implications, particularly regarding immune checkpoint inhibitor therapy [2,3].

Platinum-based chemotherapy, typically carboplatin with etoposide, remains the mainstay for systemic treatment, sometimes combined with surgery, radiation, or immunotherapy [2,8]. Our patient’s regimen, which transitioned from carboplatin/paclitaxel to carboplatin/etoposide with supportive care, mirrors these approaches. Despite aggressive multimodal therapy, outcomes remain poor. Prior studies have reported median survival of less than one year in most cases [2], with frequent extrauterine disease at diagnosis and limited responses to treatment [3]. Similarly, small-cell neuroendocrine carcinomas of the endometrium have been associated with rapid recurrence despite combined-modality therapy [7].

Our case is further distinguished by symptomatic spinal involvement requiring urgent neurosurgical intervention, underscoring the neurologic complications that may arise from early hematogenous dissemination. Elevated tumor markers (CA-125, CEA, and CA 19-9) in our patient also reflected extensive disease, reinforcing the need to consider NEC in rapidly progressive gynecologic malignancies with atypical presentations.

Endometrial NECs may appear in pure form or mixed with adenocarcinoma or sarcoma, and rare variants such as primitive neuroectodermal tumors have also been described in the uterus [4,9]. Fewer than 50 uterine cases have been reported, though small-cell carcinomas of the genital tract more commonly arise in the ovary [10-12]. Prognostic indicators include metastatic disease at presentation, pelvic or central involvement, older age, larger tumor size, elevated lactate dehydrogenase, and poor response to chemotherapy [10,11]. Given the rarity of reported cases and absence of standardized algorithms, management remains individualized and typically multimodal, integrating surgery, chemotherapy, and radiation [8]. Our patient’s course highlights the importance of early multidisciplinary coordination to manage both oncologic disease and symptom burden.

## Conclusions

Endometrial NEC represents a highly aggressive and diagnostically challenging malignancy with limited therapeutic options and dismal outcomes. Our case underscores how atypical initial presentations, such as acute neurologic deficits from spinal metastases, can obscure timely recognition and delay treatment. Accurate histopathologic evaluation, including immunohistochemistry and careful differentiation from poorly differentiated or dedifferentiated endometrial carcinoma with neuroendocrine features, remains critical for establishing the correct diagnosis. While platinum-based chemotherapy remains the cornerstone of management, optimal care requires early multidisciplinary involvement to address both systemic disease and symptom burden. Broader reporting of rare cases such as this is essential to refine diagnostic criteria, expand collective clinical experience, and ultimately inform evidence-based guidelines for future management.
